# Psychological Treatments for Hyperactivity and Impulsivity in Children with ADHD: A Narrative Review

**DOI:** 10.3390/children10101613

**Published:** 2023-09-27

**Authors:** Shayan Sadr-Salek, Andreia P. Costa, Georges Steffgen

**Affiliations:** 1 Service Psychologique, Solidarité Jeunes asbl—Haus 13, 48, rue Victor Hugo, L-4140 Esch-sur-Alzette, Luxembourg; shayan.salek@s-j.lu; 2 Institute for Health and Behaviour, Department of Behavioural and Cognitive Sciences, University of Luxembourg, Campus Belval, MSH, 11 Porte des Sciences, L-4366 Esch-sur-Alzette, Luxembourg; georges.steffgen@uni.lu

**Keywords:** ADHD, hyperactivity, impulsivity, treatment, narrative review

## Abstract

Treatment of the ADHD types (hyperactive-impulsive, inattentive, and combined) in children has rarely been studied separately, although their prognostic courses differ widely. In addition, data show that improvements in hyperactivity/impulsivity are hard to achieve. Thus, we focused on treatments tailored to hyperactivity/impulsivity. We examined meta-analyses and systematic reviews within the inter- and intra-individual treatments and found that psychoeducation and training for parents, school-based interventions, reinforcement strategies, and neurofeedback consistently showed small to moderate effect sizes in reducing hyperactivity/impulsivity in children. Conversely, emotional self-regulation, social skills, and cognitive trainings showed unsatisfactory results. In summary, we found that the quality of usual care can be surpassed when the designated interventions are purposefully combined into a multimodal treatment program.

## 1. Introduction

### 1.1. Clinical Picture

Attention deficit hyperactivity disorder (ADHD) is one of the most common mental disorders in childhood [1,2]. ADHD is characterized by the three core symptoms of inattention and/or hyperactivity and impulsivity. In short, these symptoms are present at an abnormal level (in relation to age and developmental stage), occur across situations, and cause significant distress and/or limitations in social, educational, or occupational functioning [3,4]:

1. Inattention is defined by forgetfulness, distractibility, and careless mistakes. Moreover, concerned persons commonly seem to have difficulties listening when they are spoken to, finishing what they started, and organizing themselves as expected. They also often avoid or refuse to do their homework and frequently lose their toys and belongings;
2. Hyperactivity is characterized as fidgeting and not being able to sit still, respectively, by running and climbing around in inadequate situations. Moreover, hyperactive children may not be able to play in a quiet way or, at least not over an extended time span. Moreover, excessive motor activity often cannot be managed by the social environment and direct orders;
3. Impulsivity is regarded as interrupting others or not being able to hold back answers, thus bursting into other people’s conversations. Impulsive children talk excessively and often change the conversation subject. Consequently, these children are very impatient, and they find it difficult to wait their turn.

### 1.2. Epidemiology/Prevalence

The prevalence of ADHD is age-dependent and varies with the diagnostic criteria and sources of information used to assess symptomatology. In childhood and adolescence, the prevalence found in international epidemiological studies is around 5.3% worldwide, with no significant differences internationally [5]. For instance, in Germany, the frequency of parent-reported diagnoses of ADHD is about 5%, according to the KiGGS study of the Robert Koch Institute [6]. For adulthood, a prevalence of 2.5% was found in a meta-analysis of six studies based on the DSM-IV criteria [7]. Moreover, Polanczyk et al. [8] could illustrate that the prevalence has not changed over the last three decades on a worldwide basis.

In population samples, the prevalence ratio between boys and girls varies around 3:1, whereas in clinical samples, the prevalence ratio between boys and girls varies around 6:1 [9]. Scahill and Schwab-Stone [10] found, between the ages of 4 to 16 years, a prevalence rate of 9% for boys and 3% for girls. In the same way, girls are less likely to have comorbid aggressive symptoms [9]. In addition, Schlack et al. [6] showed that higher prevalence rates of ADHD are correlated with lower socioeconomic status and that living in big cities is linked with higher prevalence rates of ADHD compared to living in the countryside.

Moreover, prevalence estimates for ADHD types vary considerably across studies, and no consensus has yet been found on which type is more prevalent. Depending on the study, the prevalence rates for the hyperactive and impulsive type range from 0.7% to 2.77%, while the prevalence rates for the inattentive type range from 0.3% to 3.2% [11,12,13,14]. Concerning comorbidities, 30–50% of all ADHD cases meet the criteria for oppositional defiant disorder, 20–30% for learning disabilities, 20% for anxiety disorders, 15% for depressive disorder, and 10–20% for tic disorder [9].

### 1.3. Etiology

The conditions for the development of ADHD are extremely diverse as multiple interacting factors are involved in the pathogenesis of ADHD. Genetic predispositions and pre-, peri-, and early-postnatal environmental influences that affect structural and functional brain development play a central role [15].

Research on molecular genetics has shown that particularly dopamine and serotonin seem to be involved in the pathogenesis of ADHD. Heightened dopamine transporter concentration and reduced dopamine concentration in the synaptic gap have been found to be associated with ADHD symptoms [16]. Furthermore, findings from both behavioral and molecular genetic studies indicate that genetic as well as non-genetic factors contribute to the development of ADHD. Faraone et al. [17] provide a review of family, twin, and adoption studies and found convincing evidence that genes play an important role in transmitting vulnerability to ADHD. This is most apparent in twin studies, which value the heritability of ADHD at 0.76. Moreover, Fortier et al. [18] present genetic evidence for the implication of the hypothalamic–pituitary–adrenal axis in ADHD, which may serve as an important mediator in the pathogenesis of the disorder. Furthermore, the severity of ADHD was found to be higher in children with parents affected by ADHD than in children whose parents were non-affected [19]. 

In addition, parental ADHD was linked to a higher likelihood of being diagnosed with the combined ADHD type and a lower chance of being diagnosed with the inattentive type alone [19]. Apart from this finding, there is only scarce evidence on the different backgrounds of the ADHD types [4].

Intriguingly, Tully et al. [20] have found that early births or low birthweight significantly increase the risk for later development of ADHD. They also found that maternal warmth served as a protective factor, counteracting birthweight or birth time as risk factors. Furthermore, infections, toxins, head traumas, and diet need to be further studied in the multifactorial pathogenesis of ADHD [4,9].

Moreover, ADHD is frequently paired with cognitive and executive deficits that are related to diffuse brain abnormalities. The anterior cingulate gyrus and the dorsolateral prefrontal cortex are significantly smaller in ADHD patients. These changes are thought to explain deficits in purposeful behavior. In addition, activity in the frontostriatal region is similarly impaired in these individuals, as shown by fMRI measurements. An understanding of these pathophysiological mechanisms is crucial so that interventions can be targeted to them [21,22].

Additionally, a range of psychosocial factors are commonly discussed in the literature, which have been shown to moderate the intensity of children’s hyperactive and impulsive behaviors. In general, a genetic disposition is considered the main causative variable of ADHD. However, unfavorable conditions in the family and school can lead to an increase in negative interactions in the environment, which, in turn, can amplify symptoms of ADHD. Moreover, the family is the first environment in which children learn rules and boundaries. In families where this process is not successful, the adoption of rules becomes progressively more problematic throughout life and leads to increasingly harsher consequences [4]. In this regard, Fenesy et al. [23] examined childhood ADHD symptoms and negative parenting styles as predictors of prospective changes in social problems over a four-year period. Initially, families of children with and without ADHD were closely examined, encompassing observed positive and negative parenting styles, youth ADHD symptoms, and multiple modal ratings of youth social problems. Along with other research, their findings suggest that psychosocial and educational factors (e.g., authoritarian parenting style, reinforcement through inconsequent parenting, absence of consistent rules, shortage of concrete action plans for parents and children, lack of school interventions, inadequate social skills, poor cognitive stimulations, imbalance of reward and punishment, etc.) are highly implicated in the underlying vicious circle that feeds hyperactive and impulsive symptoms in particular and moderates the severity of the disorder over the developmental period in children [3,4,23,24,25,26,27,28,29,30].

### 1.4. Diagnostic

The criteria of the ICD-11 or the DSM-5 classification systems must be fulfilled to diagnose ADHD. Both classification systems specify three types, namely the predominantly inattentive type, the predominantly hyperactive/impulsive type, and the combined type. According to the American Psychiatric Association [1], diagnosis of the inattentive type is made if the sub-points for the hyperactive and impulsive characteristics are not fulfilled; diagnosis of the hyperactive/impulsive type is made if all criteria are met except the sub-points for the inattentive type. Diagnosis of the combined type is given if all diagnostic criteria are met. 

The diagnosis also requires the presence of at least moderate functional impairments. The symptoms of inattention or hyperactivity and impulsivity and the resulting functional impairments should occur in several areas of life, usually before the age of 12 [21]. If the symptoms only occur in one area of life (e.g., only at school/work, only in the family), this may be an indication of another mental disorder (e.g., adjustment disorders due to stress in the family or at school) that should be clarified by differential diagnosis [4]. Thus, ADHD diagnostics should always be multidimensional. This means that the affected persons themselves, relatives, or close persons should be interviewed. It also means that a variety of examination methods should be used, such as clinical interviews, questionnaires, behavioral observations, and objective tests [4]. An anamnesis should help to understand the history of the person and to consider critical life events. Differential diagnostics should consider especially oppositional defiant disorder and learning disabilities [9]. In addition, an intelligence test can further clarify the strengths and weaknesses of the person, and intervention methods can be adapted to the present intelligence profile, especially since 50% of ADHD patients have noticeable executive dysfunctions [31,32]. To complete the diagnostic evaluation, medical and neurological examinations are necessary to assess the underlying brain activity and functioning [33]. ADHD may take different manifestations depending on the person’s age, which can reflect differences in brain regulatory systems [34,35,36]. Therefore, an overarching assessment of the child’s functional maturity is needed to correctly diagnose ADHD and define a treatment plan [37].

### 1.5. Prognosis

The prognosis of ADHD varies according to the age of the person concerned. ADHD symptoms are found to persist into adolescence and can affect social and academic areas of life. A total of 40% of patients continue to show symptoms into adolescence, while 25% are diagnosed with an anti-social disorder at the same time, and recent research points to an apparent trend in the increase in the prevalence of ADHD among adults [38,39]. Nevertheless, there is also an important long-term trend in which ADHD patients’ symptoms decrease by about 50% in adulthood. Typically, 50% of patients “outgrow” ADHD, including through treatment, and 25% do not require treatment until they reach adulthood. Commonly, two theories are discussed to explain these developments. First, stimulants help improve frontal lobe development over time, and second, adults with ADHD often choose occupations that do not require sustained attention. As adults, these patients are then able to achieve their educational and occupational goals [40]. 

ADHD treatment, on the one hand, has been found to be effective in improving the symptoms of oppositional defiant disorder and conduct disorder. Likewise, the risk of substance use has been shown to be considerably reduced. Untreated ADHD, on the other hand, can lead to persistent dysfunction and devastating consequences, including long-term disability, a heightened risk of substance abuse, and an increase in car accidents [21,41,42,43,44]. In this regard, the symptom of inattentiveness, which can hinder the full understanding of the consequences of numerous actions, is hypothesized to be partly responsible for various crimes, such as theft, drug use and sale, robbery, and even burglary [45]. In contrast, individuals suffering from the hyperactive/impulsive type of ADHD tend to disrupt their routines, lose touch with their friends and families, and engage in risky behaviors even if they understand the consequences of their actions better than those suffering purely from inattentiveness. Those with the hyperactive/impulsive type, conversely, commit reckless crimes that lead to arrests and convictions to a higher degree than those with the inattentive type. These crimes relate to theft and especially assault, but not drug trafficking, as the latter, presumably, requires forethought [46]. Thus, there are clear predictors of the differences in the prognosis, which become apparent by evaluating the crimes committed by people suffering from the different types of ADHD alone [46].

### 1.6. Negative Consequences of ADHD over the Life Span

Children with ADHD suffer significantly from their disorder and face various consequences throughout their school years and beyond [46,47]. First, the school curriculum poses a challenge that children cannot follow due to their hyperactivity/impulsivity, which is often accompanied by oppositional behavior or due to their inattention and absent-mindedness [9]. This often leads to poor school grades and/or a conflictual school environment, which not infrequently results in the affected children beginning to devalue the school itself [48]. As a result, defiant and stubborn behavior toward any kind of schooling repeatedly occurs, leading to children performing significantly worse in school compared to their peers [47,48].

Thereafter, transitioning to puberty with ADHD is a major task. Classic developmental tasks such as detachment from the parental home or the special importance of identity development are influenced by ADHD and place a particular burden on adolescents with the disorder [49]. The influence of family members and peers should also be mentioned at this point. For example, a negative parenting style can further increase the risk of developing an affective disorder [50,51]. This should be prevented and addressed by activating resources within the family. In addition, peers may predispose adolescents with ADHD to develop strong externalizing behavior disorders (e.g., aggression, dissocial behavior) and substance abuse, as they are prone to these behaviors from the beginning. These behaviors are also associated with the early onset of conduct disorders, which, in turn, points to the need for early intervention in children [46].

Ultimately, in adulthood, the likelihood of being unemployed, addicted to drugs, implicated in accidents, or involved in crimes and punished by the justice system is much higher [1,43,44,46,52], which also places a significant economic burden on the public sector [53]. These negative developments represent a major loss for those affected, their families, and society. Social integration plays a critical role throughout the course of the disorder and poses a considerable psychological strain on all other areas of life [46]. Although many interventions attempt to alleviate the symptoms of ADHD, no major breakthroughs have been made in this field to proactively reduce prevalence rates and/or to address the underlying neuropathological mechanisms [52].

Although the ADHD literature focuses almost exclusively on evidence and interventions of the ADHD inattentive and hyperactive/impulsive types combined, the current paper sets itself apart by considering the conceptual gap that the diagnosis of ADHD entails, as there are substantial differences between the types. In short, the inattentive type is defined by distractibility, carelessness, frequent daydreaming, and forgetfulness, while the hyperactive/impulsive type stands out with restlessness and fidgetiness during any calm activities, interruptions, and disturbances of other people and often getting into trouble [54]. These differences within this psychopathology commonly lead to divergent developments and consequences and, hence, require an individually tailored and modular treatment plan. The distinction between the two types and their implication for treatment is especially important for children, for whom there is a clear distinction between the presentation of the inattentive type and the hyperactive/impulsive type, while in adults, this distinction has been found to be less clear [55]. Moreover, recent advances in psychotherapy as a field are also considering modular therapeutic approaches to treat the individual patient’s needs rather than relying only on predetermined tools that are used in a general way to treat a given diagnosis [56,57]. Therewith, this paper challenges not the diagnostic conception of ADHD but rather examines the implications for psychological treatment. Specifically, this paper seeks to highlight the hyperactive/impulsive pathway since this pathway differs too much from the inattentive one, and addressing both exceeds the scope of this work [45,46]. Additionally, as the expression can vary significantly between the ADHD types, practitioners can benefit from more explicit guidance on best practices depending on the different types. This aspect, however, has only been addressed very sparsely within the ADHD literature.

Consequently, the main purpose of the present review is to critically examine the current state of knowledge concerning the psychological treatments of the hyperactivity and impulsivity dimension of ADHD in children and adolescents. For this purpose, after having explained the clinical picture, epidemiology, etiology, diagnostics, prognosis, and negative consequences of ADHD, we will continue by describing the interventions for hyperactivity and impulsivity in children with ADHD and the associated results.

## 2. Methods

### 2.1. Search Strategy

Boolean search with combinations of “ADHD”, “attention deficit and hyperactivity disorder”, “hyperactivity”, “impulsivity”, “psychological treatment”, and “pharmacotherapy” was performed in the electronic databases PubMed, PSYNDEX, and Google Scholar between 1967 and 2022. The cross-references of the articles were also checked. Additionally, the filter options with meta-analysis, randomized controlled trials, or systematic reviews were chosen depending on the availability of the research junction and the area of interest.

### 2.2. Study Selection

Peer-reviewed studies carried out in children with ADHD and published in English or German were selected for the current review. Case reports and animal studies were excluded from the current selection.

### 2.3. Data Extraction

The studies were extracted using the above-stated criteria and were categorized into different themes such as clinical description, epidemiology, etiology, diagnostics, prognosis, and interventions. Moreover, because of the focus of this paper on the hyperactive/impulsive type, the effects of the discussed treatments on inattention in children with ADHD were not considered and are thus not reported in the following sections.

## 3. Interventions

Numerous interventions attempt to alleviate ADHD symptoms. These interventions range mainly from psychosocial therapies to bio- and neurofeedback therapies, nutritional and herbal therapies, and psychopharmacotherapy [2,58,59,60]. In this regard, this review mainly investigates the state-of-the-art psychological treatments for hyperactivity and impulsivity in children with ADHD and, hence, focuses on the psychosocial intervention techniques, including neurofeedback, while drawing comparisons with psychopharmacotherapy. Moreover, the reported psychosocial intervention techniques can be subdivided into the inter-individual versus the intra-individual domains. This enables the present review to classify the indicated techniques concisely into essential subcategories, which furthermore provide information about the basis on which the interventions operate, either in the social setting or on the individual child. Moreover, this paper aims to provide an integrated and modular treatment overview. Thus, the following sections will focus specifically on parent training, psychoeducation, school interventions, and social skills trainings as inter-individual therapeutic modules and on cognitive skills, emotional self-regulation, and neurofeedback as intra-individual therapeutic modules with the aim of increasing children’s hyperactivity and impulse control. This selection has also been drawn in accordance with the work of van der Burg et al. [42], who have systematically analyzed the most relevant therapeutic components and distilled further evidence in which ways these components can be improved to increase the quality of ADHD therapy.

### 3.1. Inter-Individual Interventions

#### 3.1.1. Parent Psychoeducation and Parent Skills Trainings

Psychoeducation is a therapeutic element that mainly teaches information about psychological disorders and their treatment. The aim of these psychoeducational approaches is to promote symptom recognition, ensure treatment participation, improve treatment adherence in both psychosocial and pharmacological domains, and teach coping skills to patients and their families [61]. Accordingly, in some studies, sessions include didactic presentations, discussions, detailed written instructions or programs, as well as guidance and skills training for parents [61,62]. Additionally, psychoeducation has frequently been proposed in combination with behavioral interventions, such as self-control learning [63], classroom management techniques [64], or parent and family counseling [65]. Research has also shown that pharmacotherapy with psychoeducation leads to greater improvements in symptoms and family relationships, among other outcomes, compared to pharmacotherapy alone [66,67]. 

Ferrin et al. [68] developed a comprehensive 12-session long psychoeducation program for families of children and adolescents with ADHD and used this program in a randomized controlled trial. The program content ranged from an overview of core symptoms, etiological factors, comorbidities, prognosis, pharmacological treatments, cognitive behavioral therapies, and neurofeedback to several sessions addressing problematic everyday situations. The psychoeducation group consisted of 7 to 10 families who attended six two-hour sessions per week. During the last three sessions, they were briefly introduced to a range of behavioral strategies to manage ADHD symptoms and reduce challenging behaviors. For the control group, treatment was continued with their usual medical doctor. In their study, Ferrin et al. [68] found statistically significant treatment–time interactions for total ADHD symptoms and a significant reduction in parent-reported hyperactivity/impulsivity with a medium effect size compared to treatment as usual. Consequently, comprehensive psychoeducation programs should be considered as a valid and complementary approach in the treatment of ADHD. Thus, clinical guidelines for the treatment of hyperactivity/impulsivity in children with ADHD suggest that treatment should include psychoeducation as an important component [69,70].

In contrast to psychoeducation, parent skills trainings are psychosocial interventions that train parents in cognitive and behavioral techniques that they can use to manage their children’s difficult behavior. The programs vary in their nature and content, but usually, they are manual-based and may include discussion sessions and the use of videos and role-playing. In addition, ADHD parent training generally includes psychoeducational components about ADHD and explains how the presence of ADHD affects a child’s functioning and behavior. Typically, programs are conducted in groups of parents and comprise 10 to 20 weekly sessions lasting one to two hours. They cover a range of topics, including the nature of ADHD, positive reinforcement skills (e.g., paying careful attention to appropriate behavior and ignoring undesirable behavior), reward systems, stimulus control techniques, the use of time-outs, working with teachers, and planning ahead for problems [71].

Zwi et al. [30] described in their meta-analysis evidence that parent skills training has a positive impact on the general behavior of children with ADHD. Similarly, the researchers discovered that it reduced parental stress and increased parental confidence. However, they did not find statistically significant improvements in the child’s hyperactive/impulsive behavior. Similarly, van der Oord and Tripp [72] found that parent training is moderately effective in reducing oppositional behavior and improving parenting practices, but it did not reduce ratings of ADHD symptoms, including hyperactive/impulsive behaviors, when independent evaluators were blinded. Moreover, the training effects appear to disperse in long-term follow-up evaluations. Nonetheless, van der Oord and Tripp [72] recommend parent and teacher training as a psychosocial, evidence-based treatment for school-aged children.

In summary, extensive psychoeducation programs are comparable to parent training programs and can even outperform parent trainings when the chosen content within the programs provides a comprehensive treatment rationale for the hyperactive/impulsive symptoms of children with ADHD.

#### 3.1.2. School Interventions

Teachers of children with ADHD are often in challenging positions. They must demand from the concerned child a behavior that is particularly difficult for the child, i.e., to sit quietly for a long period and to concentrate on the content of the lesson. Moreover, there are often several problematic children in the same class. Hence, teachers frequently find themselves in a vicious circle when trying to meet the individual needs of children with ADHD. The teacher blames the child, ends up getting angry, and does not know what to do. However, if the child occasionally manages to comply with the teacher’s demands, the teacher is pleased that he or she can continue with the lesson undisturbed and does not take the opportunity to praise the child’s behavior. This exemplifies how schooling can be exceptionally stressful for children with ADHD, their teachers, and the whole class [28,64].

Therefore, Miranda et al. [64] studied the effectiveness of a multicomponent program that was taught to teachers and implemented by them in a classroom. The program included components such as general information about ADHD, learning principles, positive reinforcement, the Premack principle, and cognitive behavioral techniques such as self-control and self-instructional procedures. Dependent measures included neuropsychological tasks, behavior rating scales for parents and teachers, direct observation of classroom behavior, and academic records of children with ADHD. Teachers were trained in the use of behavior modification techniques, cognitive behavioral strategies, and classroom management strategies for 29 children. The remaining 21 children formed the control group of a total of 50 children who participated in the study. Parent and teacher ratings showed improvements in the primary symptoms of hyperactivity/impulsivity and behavioral difficulties associated with ADHD. The results also show that academic performance and classroom behavior observations improved and that teachers were more familiar with strategies they could use to meet the educational needs of the children more effectively.

Moreover, Richardson et al. [28] examined the effectiveness of non-pharmacological school-based interventions for students with ADHD and the conditions that may augment or limit their effectiveness. In their systematic review, 54 controlled trials met the inclusion criteria. Positive significant effects for various symptoms and school outcomes were observed in 36 of 39 meta-analyzed randomized controlled trials. Mean weighted effect sizes ranged from very small to large for improving hyperactive and impulsive behavior; thus, considerable heterogeneity in effect size estimates was found between studies. Furthermore, moderator analyses could not clarify which intervention characteristics were associated with which degree of effectiveness.

In conclusion, DuPaul et al. [73] argue that school-based interventions are essential components of a well-integrated treatment plan for children with ADHD. Such interventions are effective complements to pharmacotherapy and family-based interventions, particularly regarding direct effects on academic and behavioral outcomes in the school classroom. Moreover, DuPaul et al. [73] state that therapy plans should include a balanced combination of proactive (i.e., antecedent-based) and reactive (i.e., consequence-based) behavioral interventions to best manage hyperactive and impulsive outbursts.

#### 3.1.3. Social Skills Trainings

Social skills are made up of complex relationships involving multiple aspects of cognition, emotion, and behavior. Social skills trainings, designed specifically to address the characteristics of ADHD, intend to improve inter-individual and communication skills and reduce social difficulties [74].

Children who display impulsive-aggressive behaviors often have problems expressing their needs and wishes appropriately and subsequently end up with social difficulties. They also often resort to inappropriate problem-solving strategies or do not take the time to think about a suitable solution. Not infrequently, dysfunctional communication and action patterns lead to a reinforcement of inter-individual problems and existing conflicts. As a result, children and adolescents with aggressive behaviors experience rejection, which, in turn, reinforces their own mistrust and the perception that others are hostile toward them. An important component in the psychotherapeutic treatment of children and adolescents with hyperactive and impulsive issues is, therefore, the development of social skills. By building socially appropriate assertiveness and adaptive skills, children learn alternative and socially appropriate strategies for communication and interaction situations in which conflicts often arise [27].

To train these situations, it is recommended to practice concrete solutions by means of role-play in addition to exercises and games that explain social situations and improve children’s theory of mind. This also provides the opportunity to jointly analyze and, if necessary, improve practical implementations using video recordings. The transfer of skills for children with hyperactive and impulsive issues into everyday life is mainly achievable by extending the setting. For instance, by training in a group setting, integrating people from everyday life (e.g., through the participation of siblings in individual sessions), and educating and informing educators or teachers about certain strategies practiced in therapy so that they can also observe, support, and give feedback on the corresponding implementation in everyday life [72].

Social skills programs focus on varying facets but typically on problem solving, emotion control, and verbal and non-verbal communication. Generally, these training programs concentrate on helping children learn how to read the subtle hints in social interactions, such as learning how to wait for their turn, recognizing when to change the subject during a discussion, and being able to detect the emotional expressions of others. Children can be shown how to practice matching their verbal and non-verbal behavior in their social exchanges. Such trainings could also include efforts to change children’s cognitive evaluation of the social world. Thus, social skills trainings also include knowledge about the social norms, social rules, and expectations of others [74].

Social skills trainings are often conducted in groups and are comparatively short-term programs. The trainings usually span between 8 and 12 weeks, while each group session usually lasts between one hour and 90 min. Therapy sessions can range in frequency from a couple of times a month to several times a week [74].

Despite all of the aforementioned research, Evans et al. [75] reported that even though social skills trainings are a common intervention approach, their findings suggest that social skills trainings have limited effectiveness in reducing hyperactive and impulsive behavior, at least when delivered in traditional clinical settings. Mikami et al. [27] accentuate these findings by highlighting two promising directions in which social skills trainings can be adapted to enhance their effectiveness for hyperactivity/impulsivity. First, social skills trainings should entail stronger reinforcement and reminders of appropriate social behavior in real-life peer situations, which also requires that such reminders can be accepted prior to a potential outbreak. Second, the intervention effects increase when the peers are engaged to be more socially inclusive and supportive of the affected children with ADHD. However, to date, this pathway has been under-researched in the literature.

### 3.2. Intra-Individual Interventions

#### 3.2.1. Cognitive Trainings

ADHD, particularly the hyperactive/impulsive type, is associated with an increased tendency to react impulsively to strong emotions, which, in turn, is closely associated with deficits in cognitive control. To counteract this, cognitive trainings can be delivered through user-friendly and evidence-based PC programs. Such programs have been developed with the aim of improving mental performance. Cognitive functions such as attention, concentration, memory, visuomotor coordination, reaction and information processing speed, and impulse control are common targets of these trainings. Some trainings also claim to promote stress tolerance and mental resilience [76]. Peckham and Johnson [76] could also show significant improvements in the trained areas of working memory and inhibition tasks, as well as improvements in an inhibition transfer task. Overall, their results support the effectiveness of cognitive training interventions as a mean of reducing emotion-related impulsivity in adults.

In children with ADHD, Paul-Jordanov et al. [77] found that the effects of cognitive control through if-then plans (see [78]) modulate the event-related potential component P300 measured with a high-density electroencephalogram (EEG) and also facilitate response inhibition using a combined classification and Go/No-Go task paradigm. Moreover, this effect was comparable to the effects of methylphenidate treatment. Furthermore, methylphenidate, as well as if-then plans, modulated the P300 and improved inhibition of an adverse response in a Go/No-Go task to the same level as in children without ADHD. Thus, Paul-Jordanov et al. [77] found evidence to support their hypothesis that an impaired function of the prefrontal cortex, anterior cingulate cortex, and a respectively reduced P300 are linked to deficits in executive functions in children with ADHD and further concluded that self-regulatory strategies are a valuable alternative to drug treatment in children with ADHD. In this sense, self-regulation strategies strengthen the inhibition of unwanted reactions and help actively control hyperactive and impulsive behaviors.

Cortese et al. [25] conducted a meta-analysis of randomized controlled trials to determine the effects of cognitive trainings on ADHD symptoms, neuropsychological impairments, and school performance in children or adolescents with ADHD. Several databases were searched, and 16 studies were finally analyzed. By considering all types of trainings combined, a significant effect on overall ADHD symptoms was found. However, the score decreased significantly when evaluators were blinded. Moreover, the effects on hyperactivity/impulsivity and academic performance were not statistically significant. Hence, despite improvements in working memory, cognitive training had limited effects on hyperactivity/impulsivity, especially when assessed by blinded measures. Approaches targeting multiple neuropsychological processes could optimize the translation of the effects from cognitive deficits to everyday clinical symptoms [25].

#### 3.2.2. Emotional Self-Regulation

In everyday life, emotional self-regulation, i.e., the child’s ability to manage, direct, express, and control their emotions, is especially important when it comes to completing tasks that have unpleasant or negative connotations. Self-regulation enables the planning of actions, the concentration on these actions, and the consistent pursuit of action-oriented goals and is especially important when hindrances appear. Children who suffer from ADHD have great difficulties performing these tasks, as these children do not regulate their impulses and emotions adequately, which leaves them with a severely limited ability to self-regulate [79].

In general, the ability to self-regulate is an important predictor of a suitable level of functioning in social situations [80]. Moreover, self-observation enables children and adolescents to acquire knowledge about their own feelings and behavioral reactions and to gain insight into the motives of their personal actions. Such self-assessments are then compared with internal standards, ambitions, and expectations, which, in turn, set the basis for affective and cognitive responses [79]. Thus, deficits in self-awareness are related to poorer self-regulation and allow impulsive-aggressive behavior to manifest itself more repeatedly. Therefore, an essential component in the treatment of children and adolescents with impulsive-aggressive behavior is the sharpening of their perception of their own feelings and moods while simultaneously eliminating distractions. Ultimately, recognizing one’s own impulses and emotions is the first step in learning to control them in a competent way and further acquiring strategies for dealing adeptly with conflictual situations [81]. 

Moreover, Guderjahn et al. [79] showed that a teacher-led intervention with goal setting and if-then plans promoted self-regulation skills in children with ADHD. Herein, teachers reported significantly lower ADHD symptoms and improved self-regulation when goal intention was paired with if-then plans. However, the effects only lasted until the end of the intervention and only when the introduction of if-then plans preceded the school implementation tightly. In addition, and contrary to what was hypothesized, self-monitoring tools alone did not seem to contribute to the effects of the intervention. Nonetheless, if-then plans are an easy-to-create tool for promoting the everyday behavior of children with ADHD in the classroom and represent a promising pathway for supporting impulse control and self-regulation [79].

Complementary therewith and in accordance with most evaluations, the application of reinforcement, for which rewards and punishments are applied to redirect children’s behavior, has one of the greatest effects of all educational and psychosocial interventions [26,74,82]. In this regard, most studies directed at identifying cognitive and motivational factors involved in ADHD have examined cognitive control theories and reinforcement effects separately. Recently, the interplay between these two factors has been increasingly investigated. For this reason, Ma et al. [26] conducted a systematic and in-depth review of behavioral and neuroimaging studies that have analyzed the effects of reinforcement on inhibitory control in ADHD. The results of their meta-analyses demonstrated that reinforcement could restore inhibitory control to baseline levels in children and adolescents with ADHD, which actively contributes to controlling hyperactive and impulsive behavior. Additionally, the findings indicate that reinforcement enhances inhibitory control to a larger degree in children with ADHD compared to the controls [26]. Moreover, Storebø et al. [74] reason that emotional self-regulation is an integral aspect of resilience and that contingency management can help children effectively learn coping strategies to respond appropriately to disappointments, losses, and other distressing events.

#### 3.2.3. Neurofeedback

Neurofeedback is a computer-assisted form of behavioral therapy and a proven method for digital mental training. In neurofeedback interventions, unconsciously occurring neurophysiological processes are made perceptible to the person through feedback, and the patient can learn to control and influence the normally involuntary activities of the brain. Similar to learning to ride a bike or walk as a small child and gradually automating the movements, neurofeedback can help internalize how to behave in order to create an attentive and relaxed state [58].

On this subject, Geladé et al. [83] performed a randomized controlled trial (RCT) in which they compared the effects of neurofeedback on neurocognition with methylphenidate and physical activity as control groups. In a three-way parallel group RCT design, 7- to 13-year-old children with ADHD were randomly assigned to neurofeedback, methylphenidate, or physical activity groups over a 10 to 12-week period. Neurofeedback included theta/beta training measured at the sensorimotor cortex. The physical activity sessions were matched in time and frequency with the neurofeedback sessions. Methylphenidate was administered with a double-blind and placebo-controlled procedure. Neurocognitive performance was rated by parameters from the auditory oddball, stop signal, and visual–spatial working memory tasks. In sum, stimulants showed a better effect than neurofeedback in improving neurocognitive functions. As such, the results do not support the utilization of theta/beta training as an exclusive intervention for children with ADHD, specifically because intent-to-treat analysis of the methylphenidate group showed significantly enhanced inhibition and reduced impulsivity compared to the neurofeedback and physical activity groups. This effect was demonstrated by the increased response times and lowered error rates during the stop signal task. Regardless of the treatment, all groups showed a significant improvement in working memory.

Comparably, Van Doren et al. [29] examined the robustness of neurofeedback and control treatment effects by considering randomized controlled trials with follow-up (2–12 months) in children with ADHD. Ten studies met the inclusion criteria for their meta-analysis. Standardized mean differences in parent behavioral ratings within and between groups were calculated and analyzed. Significant medium intra-group effects of neurofeedback on hyperactivity/impulsivity were found after treatment and at follow-up. However, the non-active control conditions did not result in any significant effect size at follow-up. In contrast, active treatments (mainly methylphenidate) showed a significant medium effect size for hyperactivity/impulsivity after treatment and at follow-up. Between-group analyses also showed a little advantage of neurofeedback over non-active controls in terms of hyperactivity/impulsivity after treatment and at follow-up. Thus, compared to the non-active control treatments, neurofeedback appears to have more durable treatment effects, lasting at least six months after treatment. Overall, these studies provide a rationale for the current role of neurofeedback as an adjunct in the treatment of children diagnosed with ADHD [84].

## 4. Discussion

This review examined a range of psychosocial intervention techniques that are commonly used to treat the hyperactive/impulsive type of ADHD in children. The focus on this ADHD type allows for a straightforward analysis of the state-of-the-art intervention’s techniques without the disturbance of adapting the interventions to other symptoms that are linked, however, conceptually different, to the symptoms of interest within this paper. This sharp line further assists in reaching conclusions beyond diagnosis and prognosis and conveys a deeper understanding of treating this type specifically within the clinical practice. Furthermore, this paper incites to differentiate more between and delve into the ADHD types in the clinical setting.

The intervention techniques that have been investigated are divided into the inter-individual and intra-individual domains, containing parent psychoeducation, parent skills trainings, school interventions, and social skills trainings for the inter-individual domain and cognitive training, emotional self-regulation, and neurofeedback for the intra-individual domain.

Overall, the evidence for the discussed interventions varies considerably in terms of their effectiveness in reducing the levels of hyperactivity and impulsivity in children. In summary, both the inter-individual and intra-individual approaches appear to have positive effects on reducing symptoms of the hyperactive/impulsive ADHD type. However, within these approaches, some interventions have been found to be more beneficial than others. In the inter-individual domain, psychoeducation and training programs for parents appear to be effective in unblinded evaluations but lose much of their effectiveness when assessed by blinded evaluators. The effect sizes of the school-based interventions were found to be significant but varied between low and high in the different studies. As for social skills trainings, there is currently limited evidence of effectiveness in reducing hyperactivity and impulsivity in children. In the intra-individual domain, cognitive trainings had no transferable effects on hyperactivity and impulsivity in everyday life, but the results show a significant reduction in emotional impulsivity. Emotional self-regulation through an if-then schedule failed to maintain significant effects beyond the study period, but the effects of reinforcement strategies were strong and were able to provide comparable or even better self-regulation levels in the affected children compared to children without ADHD. In addition, neurofeedback showed medium effect sizes compared to non-active control groups in reducing impulsive and hyperactive behavior, but unlike psychopharmaceuticals, neurofeedback did not achieve the same magnitude of symptom reduction.

An additional part of the heterogeneity in results can be explained by the fact that the response to treatment depends on the intensity and quality of the treatment itself. In addition, effect sizes are compared with different control groups, which vary in all studies. However, the results also provide indications of which psychosocial interventions seem more suitable. Apart from the fact that each method offers a unique therapeutic benefit for the treated children, the interventions outlined could be combined into a multimodal treatment program and thus offer the potential to surpass the quality of treatment as usual by a considerable degree (see [63,66]).

Moreover, to complete the view of a multimodal approach in the following subsection, the transcendent effect of goal setting is discussed. Naturally, this effect needs to be subtracted from the positive impact of any other applied method. Furthermore, the combination of some effective methods will not result in an arithmetic addition of all effect sizes but rather in a slight increase in the overall effect since all methods operate largely on similar mechanisms and patterns and, hence, possess an overlying effect. Nonetheless, the general scientific evidence provides a clear consensus on the fact that a multimodal treatment approach to hyperactivity/impulsivity symptoms in ADHD represents the procedure of choice (see [63,66]).

### 4.1. Goal Setting

Setting up therapy goals represents a key element of behavior change interventions, yet it remains unclear when goal setting is optimally effective. In this regard, Epton et al. [85] have performed a meta-analysis and found a significant effect of goal setting across a range of behaviors. In their moderator analyses, goal setting was especially effective when goals were challenging, publicly specified, and/or set as a group goal. In contrast, goal setting was weaker when it involved monitoring behavior and outcomes by others without feedback. 

Rostain et al. [86] found that 88% of the publications that they analyzed contained predefined criteria for treatment response. Unfortunately, predefined criteria for normalization, remission, and/or relapse were only presented in 5%, 13%, and 4% of studies, respectively. In addition, there was a clear lack of consistency between the instruments used to measure these outcomes, as well as the criteria used to define treatment response, normalization, remission, and relapse. In this respect, therapeutic goals in treating ADHD should surpass modest reductions of ADHD symptoms to include functional remission and approach optimal treatment outcomes.

Kersting et al. [87] found that children suffering from ADHD and their primary caregivers consistently focused on the improvement in problem areas, such as the reduction in school problems, better concentration, and reduced impulsivity. Consistent with other studies, Podeswik et al. [88] indicated that school is the primary setting in which improvements are sought, and medication-related problems were of secondary importance, even though 48% of patients were receiving medication. Still, Kersting et al. [87] found that in 62% of the cases, the goals of the patients and their primary caregivers differed. Therefore, it turns out to be essential in therapy to uncover and reconcile these differences between the goals of the patient, the family, and the school.

Fiks et al. [89] demonstrated that assessing parents’ preferences and goals is useful for clinicians in understanding which treatment parents are likely to initiate for their children. Additionally, these results insinuate that, at least for achieving academic and behavioral goals, treatment initiation may be more important than the specific treatment selected in helping parents address their goals for their children. Overall, these findings further support the process of pairing parents and children with treatment that they are likely to begin and that achieve goals that are relevant for the family.

### 4.2. Critical Analysis

The evidence presented in this review is largely based on meta-analyses as well as on some comprehensive studies that evaluated the effects of therapeutic methods for treating children suffering from the hyperactive and impulsive type of ADHD. The methods studied were divided into inter- and intra-individual interventions, depending on whether the children themselves or their psychosocial environment were the main target of the intervention. 

The strength of this paper is the robustness of the underlying methodology of the reviewed studies, which have been mostly based on randomized controlled trial designs. This step ensures a bias reduction that may, in turn, affect the interpretation of results. However, there exists a range of limitations within this review that must be addressed. First, only a minority of studies included drew their conclusions from blinded ratings, which uniformly revealed smaller effect sizes compared to the results of non-blinded studies. Second, treatment as usual has frequently been used as a control group, which does not allow for the same comparative value as against a multimodal approach as a control group. In this regard, the control groups of the cited studies have been very heterogeneous, hence impeding the comparison between study results. Third, many studies have computed their results from limited group sizes, which, in turn, decreases the power of the applied tests substantially. Fourth, many studies reported an overrepresentation of comorbidities within their samples, which, in turn, possesses an undermining effect on the evaluated effect sizes. Fifth, the transferability of the effects to everyday life is unclear; this shortcoming is particularly apparent in the case of cognitive training. Besides these points, all studies suffer to some extent from a selection of information bias. However, the studies included in this review do not show any signs of a lowered quality in this regard compared to the general standards within psychosocial research (see [72]). Especially since the replication of previous treatment effects as well as the agreement of the cited meta-analyses with previous scientific findings was satisfactory.

### 4.3. Practical Implications

First and foremost, this review takes a unique stand on the diagnosis of ADHD by segregating the predominantly inattentive and predominantly hyperactive/impulsive types of ADHD in terms of treatment implementation. Even though the types have their rightful place within the overarching diagnosis of ADHD, this review draws attention to their diverse developmental trajectories. Consequently, these types should be conceptually considered differently in the clinical practice and require specific and distinctive treatment plans. Furthermore, this review provides solid arguments that an adaptive, integrative, and modular treatment approach represents the state-of-the-art treatment paradigm for the management of the ADHD hyperactive and impulsive type. Moreover, inter-individual and intra-individual interventions are considered as equally important. Beyond that, this paper mainly focuses on the effects of psychosocial interventions on the dimensions of hyperactivity and impulsivity in children.

Moreover, it is noteworthy that psychopharmaceutical treatments for the impulsivity dimension of ADHD have their rightful place and can serve as stand-alone treatments. In short, psychopharmaceuticals can also complement, enhance, or enable psychosocial treatments [59]. Santosh et al. [54] reported the superiority of medication over psychosocial treatment, as the advantageous effects of medication were greater in severe cases of children suffering from the hyperactive and impulsive type. These findings support the legitimacy of medication for this type and imply that treatment with stimulants is a primary treatment in severe cases. In contrast, other findings indicate that children with ADHD do not necessarily have a better response to medication than to psychosocial treatments and that the guidelines should, therefore, broaden their indications [90]. However, although the literature predominantly suggests that medication for ADHD ranks higher above all other psychosocial interventions in terms of behavior change, a number of reasons exist for preferring psychosocial interventions instead of medications, as the medications can bear unknown long-term effects and often possess low response rates in the clinical setting [30,54]. In addition, some children develop an intolerance to their medications, interactions with other medications are still quite unknown, and there are substantial moral and other barriers to the use of medication in young children [69]. Hence, many parents refuse to go down the pharmaceutical path, fear the emergence of side effects, or regard the medications as a means to turn their children into civilized, well-mannered children without tackling or correcting any underlying cause. These reasons highlight the fact that psychosocial approaches, even alongside pharmaceutical therapy, are necessary to help children who suffer from hyperactivity and impulsivity and to help parents and teachers in raising and educating children [30]. 

Consequently, more attention has been focused in recent years on the establishment of an evidence base for effective psychosocial treatments for ADHD [91]. Back in 1998, Pelham and colleagues [92] conducted a review of the relevant literature and concluded that behavioral parent training narrowly met the criteria for an established treatment but did meet the criteria for a probably effective treatment. A decade later, in 2008, Pelham and Fabiano [91] refreshed this review, pointing out that their findings extended the earlier review and revealed that behavioral interventions, including behavioral parent training, behavioral classroom management, and intensive summer program-based peer interventions, are backed up as evidence-based treatments for ADHD. Moreover, clinical psychological research has further demonstrated that intra-individual interventions, such as emotional self-regulation and neurofeedback, have extensive effects in reducing hyperactive and impulsive behaviors in children. These interventions have been shown to help affected children to more appropriately deal with their own feelings and moods. Additionally, their deficit in pursuing their needs in a goal-oriented way and their struggle to subordinate short-term desires to longer-term needs and goals have been lessened with the aforementioned interventions [49].

Furthermore, children with a diagnosis of ADHD tend to have multiple comorbid conditions, such as anxiety, depression, and oppositional defiant behavior, as well as relationship difficulties, so a multimodal treatment approach appears to be the most beneficial and fitting. Thus, in children with milder symptoms, behavioral interventions seem to be the first line of choice, especially for safety and user preference reasons [69,93]. 

Overall, our findings are consistent with clinical guidelines that recommend treating more severe hyperactivity and impulsivity in children with psychostimulants while offering multimodal psychosocial treatment to the children, their parents, and teachers. In contrast, for low to moderate symptom severity, a predominantly multimodal psychosocial treatment approach should be offered, using psychostimulants only when interventions do not result in clinically significant symptom reductions [9,30,69,93].

## 5. Conclusions

Clinical practice guidelines widely recommend that children and adolescents with mild to moderate hyperactivity and impulsivity should primarily be offered psychosocial treatment, while the parents should be provided with a group-based parent training program [9,30]. In the event that the symptoms do not alleviate adequately with this approach, pharmacological treatment can further be added to the psychosocial interventions [93]. In contrast, for children who present with severe symptoms, pharmacological treatment should be offered primarily [9,69]. Nevertheless, pharmacological treatment in children should at all times be part of a multimodal treatment plan that unequivocally includes a variety of interventions of the biopsychosocial model [93]. This means that participation in a parent training program in conjunction with psychological treatment for the children, such as emotional self-regulation strategies and neurofeedback, would be ideal in any case. Moreover, teachers should optimally be trained in managing hyperactivity and impulsivity in the classroom. In accordance with van der Burg et al. [42] and Evans et al. [75], this review has found that the family and school environment are prominent areas that influence ADHD characteristics within the inter-individual approach, while emotional self-regulation and neurofeedback produce promising outcomes on impulse control within the intra-individual approach. Currently, medication and/or psychological or behavioral interventions are the most prevalent advances in treating hyperactivity and impulsivity in children, whereas the proportionality of the interventions diverges extensively across treatment centers and more so across countries [94].

## 6. Outlook

First, the evidence suggests that purely psychosocial interventions for hyperactivity/impulsivity symptoms in ADHD can have positive effects in children, although considerable heterogeneity in effect sizes was found between studies. The clinical significance and the transfer of the effects into everyday life are also still unclear, as the available data often contradict each other. On that account, steps need to be taken to continuously improve psychosocial treatment outcomes for ADHD. To ensure this, emotion regulation processes such as reward and punishment and the balance between proactive and reactive behavioral interventions need to be more prominent and closely linked to the instrumental processes of psychosocial treatment. In this context, future research should investigate further into what works, for whom, and in which circumstances (see [72]).

Second, considering that this review is essentially about the treatment of hyperactivity and impulsivity in the scope of ADHD disorder, a review of current treatment options for inattention would be the work of choice to complement the present paper. Another way to complement the current gap in this research is to further explore the efficacy and effectiveness of state-of-the-art treatment options for adults. Ideally, these include the development and testing of standardized instruments to describe interventions, agreements on gold standard outcome measures to assess ADHD behavior, and the testing of a range of potential moderators alongside intervention studies (see [28]).

## Data Availability

Not applicable.
